# Small Intestinal Bacterial Overgrowth and Irritable Bowel Syndrome – An Update

**DOI:** 10.3389/fpsyt.2020.00664

**Published:** 2020-07-10

**Authors:** Will Takakura, Mark Pimentel

**Affiliations:** ^1^ Department of Medicine, Medically Associated Science and Technology (MAST) Program, Cedars-Sinai Medical Center, Los Angeles, CA, United States; ^2^ Department of Medicine, Division of Digestive and Liver Diseases, Cedars-Sinai Medical Center, Los Angeles, CA, United States

**Keywords:** small intestinal bacterial overgrowth, irritable bowel syndrome, gut dysbiosis, breath test, methane, archaea, hydrogen, hydrogen sulfide

## Abstract

Small intestinal bacterial overgrowth (SIBO) is one manifestation of gut microbiome dysbiosis and is highly prevalent in IBS (Irritable Bowel Syndrome). SIBO can be diagnosed either by a small bowel aspirate culture showing ≥10^3^ colony-forming units (CFU) per mL of aspirate, or a positive hydrogen lactulose or glucose breath test. Numerous pathogenic organisms have been shown to be increased in subjects with SIBO and IBS, including but not limited to *Enterococcus, Escherichia coli*, and *Klebsiella.* In addition, *Methanobrevibacter smithii*, the causal organism in a positive methane breath test, has been linked to constipation predominant irritable bowel syndrome (IBS-C). As *M. smithii* is an archaeon and can overgrow in areas outside of the small intestine, it was recently proposed that the term intestinal methanogen overgrowth (IMO) is more appropriate for the overgrowth of these organisms. Due to gut microbiome dysbiosis, patients with IBS may have increased intestinal permeability, dysmotility, chronic inflammation, autoimmunity, decreased absorption of bile salts, and even altered enteral and central neuronal activity. As a consequence, SIBO and IBS share a myriad of symptoms including abdominal pain, distention, diarrhea, and bloating. Furthermore, gut microbiome dysbiosis may be associated with select neuropsychological symptoms, although more research is needed to confirm this connection. This review will focus on the role of the gut microbiome and SIBO in IBS, as well as novel innovations that may help better characterize intestinal overgrowth and microbial dysbiosis.

## Introduction

Irritable bowel syndrome (IBS) is a functional bowel disorder defined by recurrent abdominal pain for at least 1 day per week in the last 3 months that is associated with 2 or more of the following: related to defecation, associated with a change in stool form, or associated with a change in stool frequency (1). Symptom onset must occur at least 6 months prior to diagnosis, but many patients suffer long-term chronic symptoms as a result of this disorder. Patients may experience various comorbidities including, but not limited to, bloating, constipation, diarrhea, incontinence, and psychological disturbances. Historically, IBS has been associated with stress and anxiety, and the brain-gut axis has been widely described as important in understanding IBS (2). Because of this, many treatments focus on antidepressants and neurobehavioral intervention (3). Although these treatments can be effective, newer studies have demonstrated more complex, organic etiologies specific to the gut that have led to novel therapeutic options. The complex network of etiologies likely represents various pathophysiological states that may be unique to the patient and include visceral hyperalgesia, intestinal permeability, immune activation, altered gastrointestinal motility, autoimmunity, and alteration of the gut microbiome. The last category, the gut microbiome, has seen an exponential growth in interest over the last few years. One manifestation of dysbiosis, small intestinal bacterial overgrowth (SIBO), is linked to IBS and will be the focus of this review.

## Definition of SIBO

While a diagnosis of IBS is based on clinical symptoms, the gold standard for a diagnosis of SIBO is the presence of ≥10^3^ colony forming units per milliliter (CFU/mL) of jejunal aspirate by culture (4, 5). However, aspiration is invasive and expensive, requiring a skilled gastroenterologist. Also, there may be sampling errors given that only a small segment of the small bowel can be aspirated, leaving the rest unexplored. Alternatively, a breath test can be used to assess microbial overgrowth in the gut. Hydrogen (H_2_) and methane (CH_4_) are exclusively produced by microbial metabolism and are exhaled on the breath (6–8). The North American consensus defines a rise in H_2_ ≥ 20 parts per million (ppm) from baseline within 90 min of substrate ingestion as positive for the H_2_ breath test, and a CH_4_ level ≥ 10 ppm at any time is defined as positive for the CH_4_ breath test (4). Two of the most common carbohydrate substrates used for the breath test are glucose and lactulose. Glucose is a monosaccharide that is readily absorbed in the proximal small intestine, whereas lactulose is a disaccharide and has more limited absorbability (9). SIBO is associated with a myriad of symptoms including, but not limited to bloating, abdominal pain, nausea, constipation, and diarrhea. A positive H_2_ breath test is diagnostic of SIBO, which has been associated with diarrhea-predominant IBS (IBS-D) and IBS with mixed bowel habits (IBS-M) (10). A positive CH_4_ breath test is indicative of methanogen overgrowth, which has been associated with constipation predominant IBS (IBS-C) (5, 11, 12). Of note, the recent SIBO guidelines have reclassified CH_4_ positive breath test as intestinal methanogen overgrowth (IMO), as methanogenesis is likely not limited to the small intestine (5, 13–15). In addition, although measurement of CH_4_ is not always included, the North American consensus and the recent SIBO guidelines both recommend that CH_4_ be measured concurrently with H_2_ during breath testing (4, 5).

## Prevalence of SIBO in IBS

**Figure 1:**
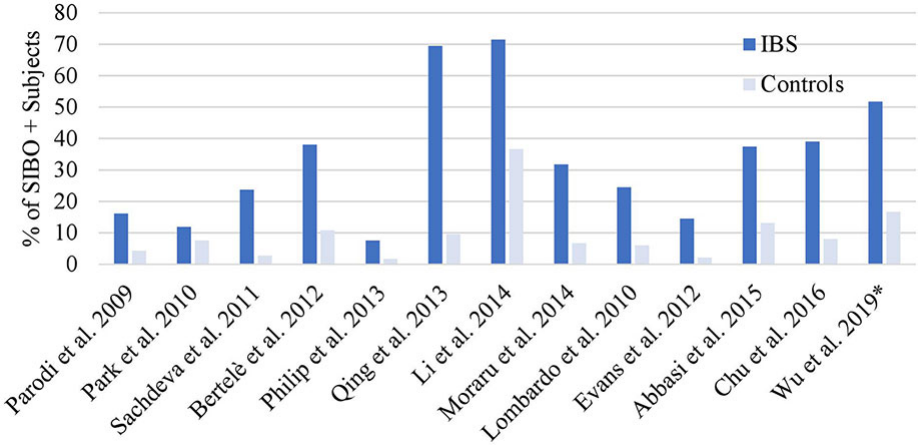
SIBO positive rates in Rome III or IV IBS. Overall SIBO is highly prevalent in IBS. *Used Rome IV definition, all other studies used Rome III (16–28).

The relationship between SIBO and IBS was described in a 2020 meta-analysis of 25 case-control studies involving 3,192 IBS subjects and 3,320 controls, that showed that the prevalence of SIBO in IBS was 31.0% (95% CI 29.4–32.6) with an OR of 3.7 (95% CI 2.3–6.0, p = 0.001) compared to controls (16). This meta-analysis included studies with healthy controls and non-IBS patient controls. When comparing SIBO rates with only healthy controls the OR increased to 4.9 (95% CI 2.8–8.6, p = 0.001). This meta-analysis included studies that had several definitions of SIBO, as well as several IBS diagnostic criteria. The Rome IV diagnostic criteria for IBS were published in 2016, and the meta-analysis did not include any studies utilizing the newer Rome IV criteria. A funnel plot evaluating publication bias showed some asymmetry, although after including only the top 15 high-quality studies with low risk of bias, the asymmetry was no longer observed. Interestingly, this increased the OR for SIBO in IBS with an OR of 4.1 (95% CI 3.0–5.6, p < 0.001). Slightly higher rates of SIBO were seen in studies that utilized breath testing as opposed to small intestinal aspiration (35.5 vs. 33.5%, respectively). The two most common breath test utilized were glucose breath test (n = 9) and lactulose breath test (n = 8). The prevalence of SIBO in IBS subjects vs controls was 62.3 vs 33.5% for the lactulose breath test, and 20.7 vs 4.4% for glucose. The association between SIBO and IBS seemed to be the strongest for IBS-D vs IBS-C or IBS-M, at 35.5% (95% CI 32.7–40.3) vs 22.5% (95% CI 18.1–26.9) or 25.2% (95% CI 22.2–28.4), respectively. Since this meta-analysis, one study utilizing the newer Rome IV diagnostic criteria for IBS has shown that the prevalence of SIBO is increased in subjects with IBS (51.7 *vs.* 16.7%, p ≤ .001) (17). **Figure 1** summarizes the prevalence of SIBO in IBS vs controls from case-control studies (n>80) using Rome III and the latest Rome IV criteria for IBS (17–29). Our analysis of these 13 studies shows that the pooled SIBO rate is significantly higher in IBS subjects than in controls (30 vs 9%, n = 2,494, p < 0.0001). The number of healthy subjects with SIBO likely reflects the complex relationship between SIBO and symptoms. A balance of specific bacterial strains with different adaptive host factors may produce asymptomatic, or mildly symptomatic patients with SIBO.

In contrast to SIBO (based on a positive H_2_ breath test), which is more strongly associated with IBS-D, IMO has been linked to IBS-C (5, 11, 16, 30, 31). Unlike H_2_, which has not been shown to directly induce diarrhea, CH_4_ has been shown to directly slow intestinal transit and causes constipation, both in humans (32) and in animal models (33). Pimentel et al. showed that direct infusion of CH_4_ in animals slows intestinal transit, and also showed that segments of guinea pig ileum exhibit increased contractile impulses when bathed in CH_4_ (33). CH_4_ appears to cause dysregulation in motility by amplifying neuronal activity in the intestine through the anticholinergic pathway (34). In humans, a study showed that the degree of constipation correlated with breath CH_4_ levels in subjects with IBS (31). Of note, for patients with constipation without IBS, the percentage of CH_4_ positive patients also correlated with constipation (35). In this study, those with decreased motility in the colon had higher levels of total and maximum CH_4_ (35). Two strict anaerobic methanogens have been isolated from the human gut, *Methanosphaera stadtmaniae* (36) and *Methanobrevibacter smithii* (37), with the latter being the predominant source of methanogenesis and associated with constipation (38). Supporting this, another study found that higher levels of *M. smithii* correlated with higher CH_4_ on breath test (12). These studies shed light on a causal relationship between methanogen overgrowth and at least a subset of IBS-C. Interestingly, bile acids are a proposed etiology of functional diarrhea and IBS-D, and have been shown to decrease methanogenesis in human feces (39).

Only a few studies have attempted to characterize the small bowel microbiome in subjects with SIBO and IBS. A North American study found that SIBO subjects had a 7–8-fold increase in *Klebsiella* and *Escherichia/Shigella* compared to non SIBO patients (40). One Indian study, on jejunal aspirate of SIBO and IBS subjects, found that 40% of subjects had *Pseudomonas aeruginosa*, 6.7% had *Acinetobacter baumannii*, 13.3% had *Acinetobacter lwoffii*, 13.3% had *Staphylococcus spp*, 6.7% had *Enterococcus faecalis*, 20% had *Escherichia coli*, 6.7% had *Enterococcus faecium*, 13.3% had *Klebsiella pneumoniae*, and 6.7% had *Streptococcus spp.* by culture (41). This study also noted an increase in *Acinetobacter lwoffii, Staphylococcus* spp., *Enterococcus faecalis, Escherichia coli*, and *Klebsiella pneumoniae* in IBS subjects with SIBO vs. those without SIBO. Similarly, a Swedish study found that IBS subjects with SIBO had a high prevalence of Gram-negative *Bacilli* and *Enterobacter* on jejunal aspirate (42). Lastly, a study from Athens found high prevalence of *Escherichia coli, Enterococcus spp*, and *Klebsiella pneumoniae* in duodenal aspirates from subjects with SIBO (43). Given the highly variable results of microbiome studies arising from different sampling locations, geography, and definitions of SIBO, caution should be exercised when generalizing these results.

## Gut Microbiome Dysbiosis, IBS and SIBO—Focus on Post-Infectious IBS Models

**Figure 2:**
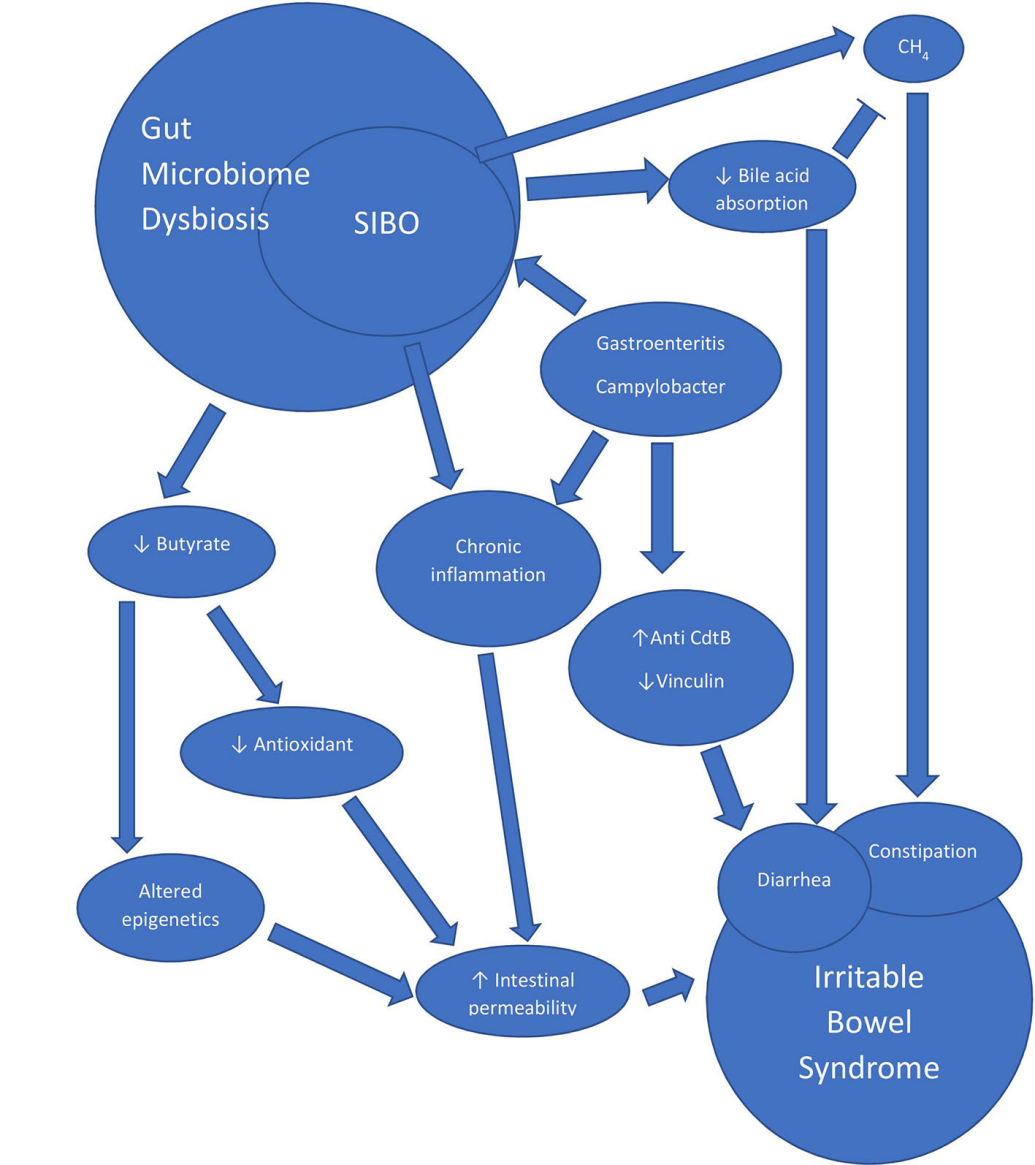
Diagram depicting selective theories on the physiologic mechanisms of IBS.

SIBO only comprises a subset of gut microbiome dysbiosis, and this review would not be complete without discussing how microbial dysbiosis in general contributes to IBS. Previous studies have shown that infectious etiologies such as infectious gastroenteritis (44, 45) and diverticulitis (46) are associated with the development of IBS, which have been termed post-infectious IBS (PI-IBS). A recent systemic review has shown that roughly 10% of patients with enteritis develop PI-IBS within the following year and the prevalence of PI-IBS seems to increase with time (47) These infections are thought to induce changes through long-lasting low-grade inflammation, an increase in intestinal permeability, and autoimmunity, ultimately leading to the symptoms of IBS (**Figure 2**). Given that roughly 10% of the population has IBS (48) and the that an estimated 10 million food-borne illnesses occur each year (49), it is possible that a significant portion of patients with IBS may have had gastroenteritis in the past that they cannot recall and may have PI-IBS. Interestingly, a mathematical model has shown that infectious gastroenteritis may contribute to a large proportion of IBS (50). Therefore, we hypothesize that studies of PI-IBS may be relevant to many subjects with IBS.

Infectious diarrhea has been known to cause intestinal permeability (51, 52), and a similar phenomenon is seen in patients with IBS (52, 53). This is thought to be partially mediated through bacterial effects on tight junctions (54). Although the mechanism(s) underlying how intestinal permeability persists after the acute infection is not entirely clear, there are many hypotheses centered on gut microbiome dysbiosis. Butyrate, a microbial metabolite, is a key player in maintaining a healthy epithelial barrier by regulating cell turnover, antioxidants, and energy maintenance in the gut lining (55, 56). Interestingly, one study found lower levels of butyrate-producing bacterial families such as Ruminococcaceae, an unknown family from the order Clostridiales, and Erysipelotrichaceae in subjects with IBS-D (57). Butyrate appears to mediate cell turnover through inhibition of histone deacetylase (HDAC), which causes apoptosis of the luminal cells (56). Interestingly, in a rodent model of IBS, inhibition of HDAC alleviated symptoms of visceral hypersensitivity (58). In addition, another candidate important in the maintenance of intestinal permeability has recently gained interest in IBS. A double-blind placebo-controlled randomized control trial in 2019 evaluated glutamine, a key amino acid that helps gut epithelial integrity (59), and found that an 8-week course significantly alleviated IBS symptoms (60). The improvement in symptoms was correlated with improvement in intestinal permeability. Interestingly, glutamine-enriched total parenteral nutrition did not improve intestinal permeability in malnourished surgical subjects undergoing gastrointestinal surgery (61), which may suggest additional mechanisms are needed to maintain the gut epithelial barrier which are not present in these subjects. Given that these subjects had poor oral intake coupled with severe gastrointestinal disease which necessitated surgery, their microbiome dysbiosis should be further investigated.

Secondly, inflammation seems to play a role in the pathophysiology of IBS. Previous studies have shown that rectal biopsies from subjects who had acute *Campylobacter enteritis* and diarrhea exhibit persistently elevated T-lymphocytes and enteroendocrine cells, and that similar elevations are seen in subjects with post-infectious IBS (52). Another study found an increase in enterochromaffin cells and T-lymphocytes in subjects with new-onset post-infectious IBS following *Campylobacter* infection compared to both subjects who were asymptomatic following infection and to healthy controls (62). The increase in lymphocytes appears to be present in the general IBS population as well, as one study found a 1.8-fold increase in intraepithelial lymphocytes in IBS subjects as compared to asymptomatic controls (63). This is not surprising since a large proportion of IBS is suspected to be due to a previous infection (50). A mouse model of post-infectious IBS developed using *Trichinella spiralis* (64) found similar results, with enterochromaffin cells being persistently elevated in the bowel long after clearance of the acute infection, as well as an increase in intraepithelial cells and a decrease in intestinal cells of Cajal in mice that developed SIBO (65). In addition, a post-*Campylobacter* rat model of IBS exhibited persistent alterations in stool consistency and in rats that exhibited SIBO, intraepithelial lymphocytes in the rectum and the left colon were also increased (66).

Autoimmunity may also play a significant role in the development of IBS as it relates to the gut microbiome. In a post-infectious IBS model, rats who were exposed to *Campylobacter* with an insertional deletion of the bacterial toxin cytolethal distending toxin B (CdtB) exhibited significantly fewer intraepithelial lymphocytes and less alterations in stool form (67). Antibodies to CdtB were found to have a high affinity to the neuronal protein vinculin, and a decrease in vinculin expression correlated with the number of times the rats were exposed to *Campylobacter* (68). Vinculin is a key cytoskeletal protein that plays an essential role in cell-matrix and cell-cell adhesion (69). In humans, numerous studies have found that these two biomarkers (antibodies to CdtB and vinculin) can differentiate between IBS-D/M (70–73) and other bowel diseases and conditions that cause diarrhea, although one study in Australia failed to show such relationship (74). A longitudinal observation of a single patient who developed IBS after an episode of infectious diarrhea demonstrated a subsequent elevation in anti-CdtB, followed by an elevation in anti-vinculin, each coinciding with symptoms (44). A thorough evaluation failed to find an “organic” gastrointestinal disorder in this patient. In humans, this pathway seems to be specific to patients with IBS-D and IBS-M but not IBS-C (71), which is consistent with findings in the rat model.

Finally, SIBO and malabsorption has also been proposed as a mechanism underlying the development of diarrhea. It is thought that deconjugation of bile salts in the upper gut induces a decrease in absorption of fat and lipophilic vitamins, leading to the production of lithocholic acid, which is poorly absorbed and thought to be enterotoxic (75). In subjects with malabsorption syndrome with and without SIBO, subjects who had SIBO exhibited significantly elevated unconjugated bile salts, acetate, lactate, and formate than those without SIBO (76).

## Microbiome-Targeted Treatment of SIBO, IMO, and IBS

There are treatments for both SIBO and IBS which target the microbiome, including treatment with a non-absorbable oral antibiotic, rifaximin. A meta-analysis which evaluated normalization of breath test in response to antibiotics for SIBO found that of the 10 studies included, rifaximin was the most common and was used in 8 studies. They found a pooled normalization rate of 49.5%, 95% CI 44.0–55.1 with rifaximin vs. 9.8%, 95% CI 4.6–17.8 with placebo (77). Rifaximin was compared against placebo in only 3 studies and had a favorable response with an effectiveness ratio of 1.97, 95% CI 0.93–4.17, p = 0.08. although this did not reach statistical significance. Four studies directly compared antibiotics to placebo and found an effectiveness ratio of 2.55, 95% CI 1.29–5.04, p = 0.03 favoring antibiotics.

Similarly, multiple large randomized controlled trials have shown that rifaximin can improve symptoms in subjects with IBS without constipation (78, 79). Currently this is the only FDA-approved therapy to treat IBS by directly affecting the gut microbiome. Unlike other medications on the market for the treatment of IBS, the effects of this treatment outlasted the duration of treatment by weeks to months. This is thought to be due to the effects of rifaximin on the gut microbiome which persist after the treatment is over. Unfortunately, a subset of subjects experience a relapse of their symptoms, but repeat dosing with rifaximin appears to be safe and effective (79, 80). The mechanism of action for this drug is not entirely clear, but it appears to induce a small, transient reduction in Shannon index as well as a decrease in the relative abundance of 7 taxa including Enterobacteriaceae, Verrucomicrobiaceae, Peptosteptococcaceae, Pasteuellaceae, Synergistaceae, Eubacteriaceae, and Enterococcaceae (81), as determined from stool samples. These transient effects are consistent with clinical observations. Whether the reductions in these taxa are directly linked to the improvements in IBS symptoms is yet to be determined. Further studies are needed to also evaluate changes in small bowel and proximal colon.

A metanalysis by Shah et al. found 7 studies which evaluated patients with SIBO in IBS and found that antibiotics relieve symptoms in 81.6% of patients. Only 5 studies reported eradication of SIBO and 93% of patients with a glucose breath test achieved normalization while 71.4% of patients who were diagnosed *via* small bowel aspirate culture reached normalization (16).

Furthermore, treatment with specific antibiotics results in decreased CH_4_ levels that correlate with improvements in constipation (82, 83). Of note, while neomycin and rifaximin can each reduce constipation in IBS-C, using a combination of both appears to be most effective (84). Currently only a small number of studies with limited sample sizes have evaluated the use of antibiotics in the treatment of IMO, and larger, multicentered randomized control trials are needed to further characterize their efficacies and potential adverse events.

Perhaps the largest clinical indication of the overlap between SIBO and IBS was demonstrated by Rezaie et al, who showed that a positive lactulose breath test predicted a favorable response to rifaximin (59.7 vs 25.8%, p = 0.002) (85) than patients who had a negative lactulose breath test. Given this finding, patients with IBS should undergo a lactulose breath test to rule out SIBO, as this may have treatment implications (86).

Finally, probiotics have also been evaluated in the treatment of IBS. A systemic review by Ford et al. (87) found that certain combinations of probiotics may be helpful in IBS, although there was significant heterogeneity between the studies with possible publication bias. In this study, combination probiotics LacClean Gold and VSL#3 had some efficacy with RR = 0.59; 95% CI 0.37–0.93 (n = 130) and RR = 0.82; 95% CI 0.52–1.30 (n = 78), respectively. Interpretation of metanalyses of probiotic studies is difficult since different strains are studied in different combinations assessing various endpoints. Many studies also have small sample sizes, making it difficult to generalize the results.

## Neuropsychological Symptoms, SIBO, and IBS

IBS patients can experience significant effects on their mental health with increased rates of comorbid psychological conditions such as stress and anxiety, as well as decreased energy and disruptions in sleep and functioning (88–90). Whether these conditions are part of the IBS-SIBO overlap remains a focus of research. A recent study by Rao et al. found that subjects with brain fog had increased rates of SIBO (91), and also found an association between brain fog and probiotic use as well as D-lactic acidosis, a condition found in patients with short bowel syndrome (92). Although the participants in this study were all referred from a gastrointestinal clinic, none were classified as having IBS, although the study did exclude subjects with “organic” gastrointestinal diseases. Furthermore, although this study was not placebo-controlled, an improvement was seen with initiation of antibiotics and the cessation of probiotics. Given this, probiotics may have subtle effects on health and despite their accessibility as an over-the-counter medication, should be taken cautiously. Similarly, in subjects with chronic fatigue syndrome, another disease which carries an enigmatic pathophysiology, erythromycin was shown to have a significant effect on sleep duration but only in those that exhibited significant effects on the gut microbiome, specifically a decrease in *Streptococcus* levels (93). It is unclear whether the promotility effect of erythromycin or its antimicrobial effect played a greater role in these findings.

Finally, one study has demonstrated a possible connection from the gut microbiome to the brain *via* the vagus nerve. In this study, mice supplemented with *Lactobacillus rhamnosus* exhibited decreased stress-related behaviors and altered expression of gamma-aminobutyric acid (GABA) receptor in the brain (94). Interestingly, this effect was decreased in rodents who underwent vagotomy, suggesting a direct neuronal effect *via* the 10^th^ cranial nerve. Unfortunately, many of these studies have methodological issues concerning blinding, open-labeled design, and small sample size, to name a few potential biases. Whether alterations in the gut microbiome can directly affect the central nervous system and whether further modification *via* antibiotics or probiotics can have beneficial effects on humans requires further research.

## Future Directions

Currently there is no consensus on the use of lactulose or glucose in breath testing (5). The lactulose breath test is limited by its potential false positive rates in patients with high motility, and the glucose breath test may not adequately detect SIBO in the distal bowel as it gets readily absorbed in the proximal small intestine (9). More validation studies are needed for standardization. As a potential solution, an ingestible capsule that can directly measure gas in the intestine is being developed. This may potentially improve the diagnostic accuracy over current methods which utilize breath testing.

There are several other innovations that may significantly influence the field of SIBO and IBS. A new study by Leite et al. has found that the use of dithiothreitol, a mucolytic, can significantly increase bacterial yield from small intestinal aspirates by approximately 3-fold (95). This will allow a more complete and thorough sampling of the small bowel which may lead to novel discoveries. As noted above, aspiration techniques can vary and this may explain why one recent study did not find any correlation between symptoms and SIBO defined by CFU/mL in a duodenal aspirate (96). Further studies will be needed to optimize sampling methods for the intestinal microbiome but until then the breath test, which has potential clinical implications and can evaluate a larger section of the bowel (85), should be utilized in the diagnosis of SIBO (4).

Interestingly, another potential marker of bacterial overgrowth on a breath test, the exhaled gas hydrogen sulfide (H_2_S), has recently been explored. H_2_S appears to have implications for multiple gastrointestinal disorders, and can relax smooth muscle and have pro- and anti-inflammatory properties (97). Singer-Englar et al. described an association between diarrhea and levels of exhaled H_2_S (98). Whether there is also an association with IBS-D (the definition of which requires pain in addition to diarrhea) remains to be seen.

Lastly, given that dysbiosis does not necessarily mean an overall overgrowth in the number of CFU and that changes in the relative abundances of specific microbial strains or taxa can cause symptoms, a reliance on measuring the overall abundance of bacteria may not be suitable in the era of 16S rRNA sequencing and breath testing.

## Conclusion

IBS and SIBO appear to be intertwined. The methods for measuring SIBO still need further optimization. SIBO and other forms of gut microbiome dysbiosis are likely responsible for some symptoms in a subset of patients with IBS and can help guide treatment options. Given its therapeutic potential, further research in IBS and SIBO is needed.

## Author Contributions

MP and WT contributed to the concept, design, and writing of the review article.

## Conflict of Interest

The authors declare that the research was conducted in the absence of any commercial or financial relationships that could be construed as a potential conflict of interest.
